# Does the Carbon Fibre Coating Reinforcement Have an Influence on the Bearing Capacity of High-Performance Self-Compacting Fibre-Reinforced Concrete?

**DOI:** 10.3390/ma12244054

**Published:** 2019-12-05

**Authors:** Krzysztof Ostrowski

**Affiliations:** Faculty of Civil Engineering, Cracow University of Technology, 24 Warszawska Str., 31-155 Cracow, Poland; krzysztof.ostrowski.1@pk.edu.pl; Tel.: +48-12-628-2313

**Keywords:** CFRP, high-performance self-compacting concrete, fibre-reinforcement, composite tube, stress-strain curve, CFRP location, reinforcement

## Abstract

This study investigated the impact of the location of a carbon fibre coated reinforcement ring (CFCRr) inside the structure of high-performance self-compacting fibre-reinforced concrete (HPSCFRC). Nowadays, cement matrix is considered as an alternative binder when reinforcing concrete structures with composite materials. Due to the plastic behavior of composite structures at relatively low temperatures when carbon fibres are reinforced with epoxy resin, the author attempted to locate carbon fibres inside a concrete structure. Thanks to this, the reinforcement will be less vulnerable to high temperatures (during a fire) and more compatible with concrete. The fibres act as a perimeter reinforcement that is compatible with the concrete mixture. The position of the CFCRr in the structure of concrete has an influence on the load capacity, stiffness and stress-strain behavior of concrete elements. The research was conducted on circular shape short concrete columns and tested under axial compression. The results demonstrated that by including CFCRr inside a concrete specimen, the maximum compressive strength decreases with an increase in the number of composite rings and a greater distance from the vertical axis of symmetry to the edge of the element. It has been proven in these studies that carbon fibres do not have good adhesive properties between CFCRr and a concrete mixture. As a result of this phenomenon, a shear surface is created, which leads to crack propagation along the CFCRr. Therefore, the presented idea of an internal CFCRr should not be used when designing new concrete structures.

## 1. Introduction

Composite materials currently belong to the group of materials commonly used in many disciplines of everyday life and also to the space that surrounds us. At least two or more materials with different chemical composition and physical properties, but with synergic micro-constituents, are involved in forming a composite material [1]. Carbon Fibre-Reinforced Polymer (CFRP) composite materials are widely used and due to their many advantages are gradually replacing classic materials. Carbon Fibres (CF) are characterized by a high Young modulus and strength, excellent fatigue properties, substantial resistance to aggressive environments, and low weight [2]. The structure of CFRP is built of two components called the reinforcement and matrix. The interphase region has specific characteristics and is a three-dimensional area between two constituents. The typical damage experienced by this specific material is delamination, fibre breakage and matrix cracking. This leads to the load carrying capacity reduction of composite material [3]. The subject of CFRP has been carefully analyzed concerning many aspects. Generally, CFRP materials fit into our landscape and are subject to environmental influences that are independent of people. It seems that the biggest challenge when using epoxy resin in CFRP materials is the effect of the temperature at which the composite element works. The elastic behavior of the epoxy resin provides changes in its energy absorption and ultimately affects the failure of the CFRP. The glass transition temperature (T_g_) is a crucial parameter in polymers, because at this specific temperature the density, rigidity and hardness in the polymer decreases. When reaching this temperature, an element becomes more elastic and ductile [4,5,6,7]. Some experiments have shown that roughness parameters of concrete elements and adhesion to epoxy resin are important parameter in terms of contact efficiency [8,9]. It has been proven that moisture diffusion has an impact on the strength and T_g_ of CFRP [10]. Appropriate adhesion between a concrete surface and CFRP laminates determines the efficiency of structures reinforced in this manner. Another aspect determining the performance of CFRP is the water absorption of laminates. Studies showing the role of impact-induced cracks in failure are also known from the literature [11]. According to the authors, cracks under an impactor propagate in a stable manner and play a big role in the ultimate failure of laminates. 

The basic structural materials are metals, in particular steel, concrete and wood. Each of these materials has different strength properties, durability and strengthening efficiency. Steel structures are characterized by high mechanical strength, good deformation and fatigue properties, and at the same time, low corrosion resistance and the need to apply surface protections. Contemporary concrete constructions, despite their high compressive strength and tightness, are also exposed to the aggressive environment, which can cause concrete corrosion. The building material known for centuries is wood. It is characterized by good compressive, tensile and bending strength, and it is a natural material with low heat permeability. As with the materials described above, a wooden structure must be protected from the effects of the environment and natural pests in order to preserve its properties for a long period of time. It is worth emphasizing that the use of composite materials can strengthen and protect steel, wood and concrete against corrosion and improve load-carrying capacity. CFRP is very effective as an alternative and effective technique to reinforce all types of structures, including old constructions, and can be seen to be a new trend in the design process. The stiffness, thickness and loading condition of CFRP affects the load-carrying efficiency of concrete elements [12,13,14]. The performance of concrete cylinders confined by CFRP has been extensively studied [15,16]. In many publications, it has been noted that with the increase of the compressive strength of concrete, the effectiveness of CFRP reinforcement decreases [17,18]. The design aspect of composite structures is very important in regarding laboratory research for industry. For confined concrete, a theoretical stress-strain model was proposed [19]. The confining pressure for reinforced concrete columns with a non-linear simulation was tested by D’Amato et al [20]. Recently, steel stirrups and fibre-reinforced polymer materials (FRP) were used to determine the analytical model proposed by Braga [21], where different types of external strengthening were considered.

In recent years, there has been an increasing number of studies concerning an alternative binder to replace the epoxy resin in CFRP composites. Due to the main disadvantages of epoxy resin—the behavior of plastic in relatively low temperatures of about 70 °C—researchers are looking for an alternative method in the lamination process, using the cement matrix. Unfortunately, this knowledge is still unfulfilled. When searching for articles in the Scopus database by entering the words ‘CFRP, cement and matrix’, only 29 results were obtained (05/09/2019). Sandrmomtazi et al. presented the influence of an inorganic and organic matrix on the behavior of FRP wrapped concrete cylinders. In this research, the authors obtained an increase in the compressive strength of specimens that were reinforced using the analyzed methods when compared to unreinforced concrete [22]. Colajanni et al., in similar research, observed a noticeable increase in the ductility and strength of analyzed specimens [23]. Al-Abdwais and Al-Mahaidi showed that modifying the content of cement-based adhesive materials has an influence on the bond properties in composite elements [24].

High-performance self-compacting fibre-reinforced concrete (HPSCFRC) is a type of concrete characterized, due to the presence of steel fibres, by high compressive strength, specific fresh concrete mixture rheological properties, and characteristic quasi-plastic stress-strain behavior [25]. Self-compacting concrete (SCC) could be considered as one of the biggest achievements in concrete technology [26]. The influence of CFRP on the behavior of normal-strength concrete (NSC), normal-strength fibre-reinforced concrete (NSFRC), HPC, and high-performance fibre-reinforced concrete (HPFRC) was noted in the literature [27,28]. When using one layer of CFRP with a 150 mm overlap, the stress-strain curve in the case of NSFRC and NSC is linear-elastic-plastic with strengthening area behavior until fracture. After the maximum stress, fragile-plastic behavior could be observed in the case of NSFRC, which was in contrast to NSC. In the case of HPC and HPFRC, strengthening does not occur. The use of fibre reinforcement inside the structure of concrete and outer reinforcements such as CFRP could significantly increase seismic resistance, which translates to the safety of people in sensitive areas [29,30]. The presence of fibres in the concrete causes non-linear behavior before the peak load [31]. Steel fibres influence on the shear and bending strength, stiffness and ductility in the HPC [32].

## 2. Research Significance 

Due to many facts such as the plastic behavior of epoxy resin in CFRP composites, a lack of interest of researchers in the subject of an alternative matrix to reinforce CFRP, the impact of aggressive environments on composite elements and structures, the need to protect CFRP that is based on epoxy resins against fire and higher temperatures, and the contemporary role of HPSCFRC that is resistant to seismic activity, the author decided to analyze the possibility of placing CFRP rings reinforced by cement mortar, which is compatible with concrete, inside the structure of circular columns made of HPSCFRC. This type of reinforcement has been called carbon fibre coated reinforcement (CFCR). One of the major current gaps concerning this topic is the determination of the stress-strain behavior of new types of concrete such as SCHPFRC reinforced with polymer materials and with cement matrix as the binder. The adhesion between composite fibres and the cement matrix can be seen as the most important issue and has a definite influence on the performance of these types of composite structures [33]. It is widely known that environmental factors have an influence on the efficiency of concrete reinforced using the FRP technique [34]. Furthermore, exposure of concrete reinforced with epoxy resin or cement-based adhesive in a high temperature leads to a decrease of strength of reinforced concrete structures with composites [35]. The motivation to address this topic was the lack of research showing the use of CF sheets in the structure of SCHPFRC. The aim of this study was to answer the major question: Does the localization of CFCR rings inside the structure of HPSCFRC have an influence on the stress-strain characteristic of this kind of composite structure?

## 3. Materials and Methods

### 3.1. Preparation of Carbon Fibre Rings

**Figure 1:**
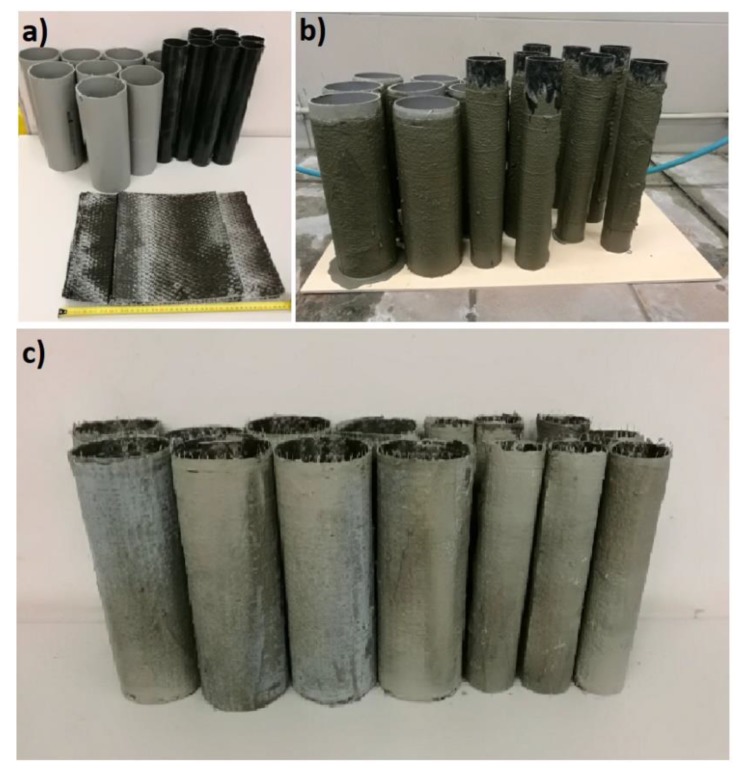
Stages of CFCR (carbon fibre coated reinforcement) ring implementation: cutted carbon fibres (**a**), carbon fibres coated with fresh cement matrix (**b**) and CFCR rings (**c**).

In order to prepare CFCR rings, Carbon Fibre Sikawrap 301c (Sika, Switzerland), polypropylene tubes and a cement matrix were used. In these studies, CFCR rings with outer diameters of 60 mm and 110 mm, a height of 300 mm and a thickness of 3 mm were prepared. In the case of all the CFCR rings, a 50 mm overlap was provided. Firstly, carbon fibre mats were cut into the appropriate length (Figure 1a). The next step involved the installation in five places of carbon fibers to the surface of pre-cut polypropylene pipes with an external diameter of 117 mm and 57 mm using a steel wire with a diameter of 0.3 mm. Then, using the cement matrix, the CF were coated with 3 mm layers (Figure 1b). After seven days, the CFCR rings were extracted from the polypropylene pipes and used in the survey (Figure 1c).

The properties of the CF, according to the manufacturer, are presented in Table 1. The proportions of the cement matrix were as follows (by mass): Cement:SikaFume:Water: Superplasticizer were equal to 100:30:40:10. The average compressive strength of the cement matrix was 62 MPa and was determined using 4 samples, whereas the standard deviation was only 1.2 MPa.

### 3.2. Concrete Mixture and Preparation of the Specimens

**Figure 2:**
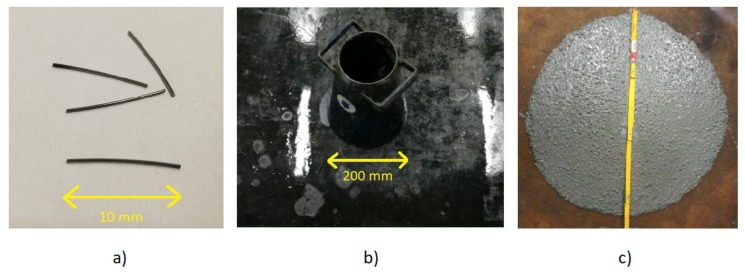
Steel reinforcement (**a**), instrumentation to Slump Flow Test (**b**) and Slump Flow for concrete mixture (**c**)**.**

The HPSCFR concrete mixture was used in the research. The constituent materials were as follows: Portland Cement type I, Sika Fume as a structural microfiller, superplasticizer Sikament FM6, diabase as coarse aggregate, sand as fine aggregate, and water from waterworks. The fibre reinforcement had the form of steel fibres with the slenderness ratio equal to 20 with outer diameter of 0.5 mm and a length of 10 mm (Figure 2a). The ultimate tensile strength of this steel, based on information from the manufacturer, was equal to 700 MPa. Details of the concrete mixture are shown in Table 2. Some fresh properties of the HPSCFR concrete mixture were determined using Slump Flow test (Figure 2b). The plastic viscosity was 12.5 s and Slump Flow was equal to 650 mm (Figure 2c), which is according to European Standards [36]. Leakage of the mortar did not occur, which proves that the concrete mixture was made properly. The sorting of components was not observed.

A total of 16 concrete specimens were tested in the study. All the specimens were 300 mm in height and had an outer diameter of 150 mm. All circular columns were divided into five groups, represented by four specimens (Table 3): plain HPSCFRC as the reference (signed C1-4), concrete with a 110 mm CFCR ring (C110-1-4), concrete with a 60 mm CFCR ring (C60-1-4), concrete with 60 mm and 110 mm CFCR rings inside the elements (C60-110-1-4) and concrete reinforced with CF sheets using cement matrix (C150-1-4). For instance, C60-110-1 is the first specimen that was made with HPSCFRC and it had 60 mm and 110 mm CFCR rings. The CFCR rings were located in the middle of the axis of symmetry and mounted in the form to the base using silicon. 

**Figure 3:**
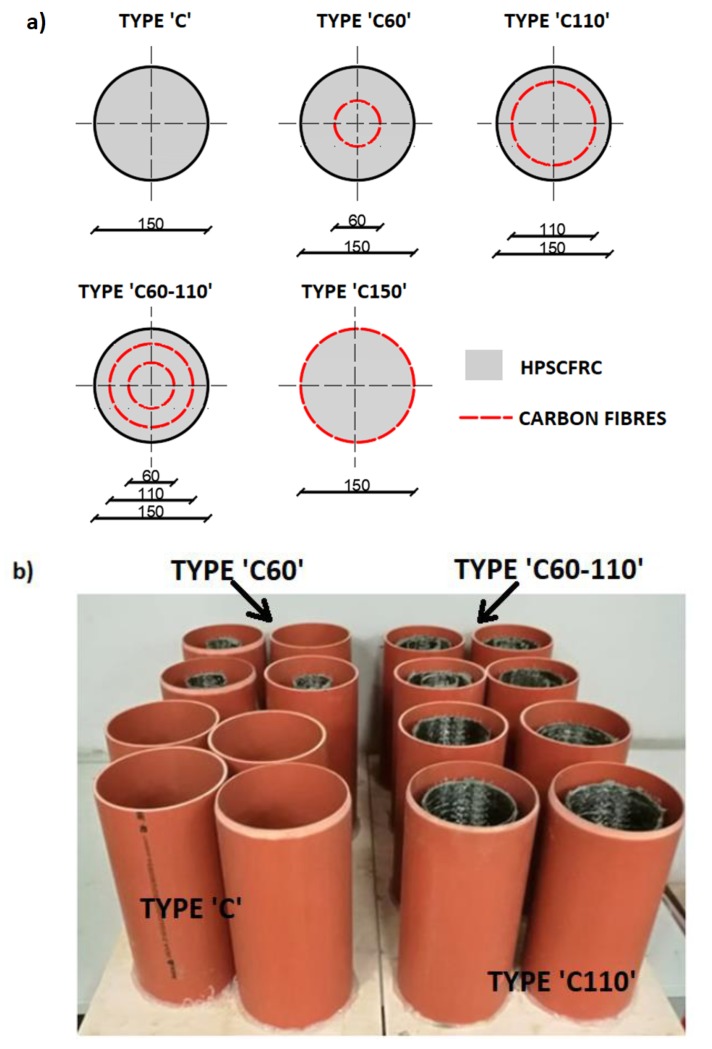
Cross-section of specimens (**a**) and CFCR rings in the polypropylene tubes (**b**).

As the form for the specimens, polypropylene tube with a height of 310 mm and an inner diameter of 150 mm was used. The concreting was performed in a continuous manner for all the specimens. On the day after concreting, the specimens were placed in a water bath and cured for 28 days. After this, the specimens were cut and their surface was grinded on their right side at a height of 300 mm. The location of the CFCR rings inside the tube is presented in Figure 3. In the case of HPSCFRC with an outer reinforcement that uses CF sheets with cement matrix, the surface of the concrete was grinded with bitumen shield to ensure better adhesive properties. Then, the concrete surface was cleaned, washed and dried. The same cement matrix, as in case of the CFCR rings, was used to connect the CF sheets with the HPSCFRC. An overlap of the outer reinforcement, equal to 150 mm, was provided. Firstly, cement matrix was applied on the surface of the concrete, then one layer of CF sheets were put on the cement composite, and finally the cement matrix was put on the CF sheets. The thickness of the cement matrix layers under and above the CF sheets for the ‘C150’ type specimens was 5 mm. 

### 3.3. Instrumentation and Testing Procedure

**Figure 4:**
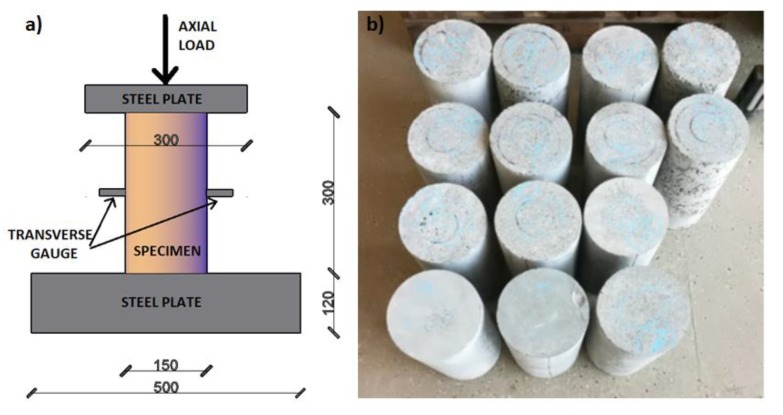
Test setup showing the LVDT application (**a**) and part of the tested specimens (**b**)**.**

The specimens were tested under uniaxial compression using a 6000-kN capacity testing machine (Walter + Bai AG, Löhningen, Switzerland). All examinations were performed in a local laboratory at an air temperature of 25 ± 1 °C and humidity of 55 ± 5%. The constant axial strain rate, equal to 4 × 10^−5^ [s^−1^], was ensured during the compression test. The axial and transverse (half way up the specimens) displacement was measured using extensometers. All the tested specimens and the test setup illustrating the Linear Variable Differential Transformer (LVDT) applications are shown in Figure 4a,b, respectively. The Young Modulus was determined in the characteristic values of stresses with 5% to 40% value of the maximum strength of each specimen. The test was in accordance with the standards [37].

## 4. Results

### 4.1. Selected Mechanical Properties of the Hardened Specimens

**Figure 5:**
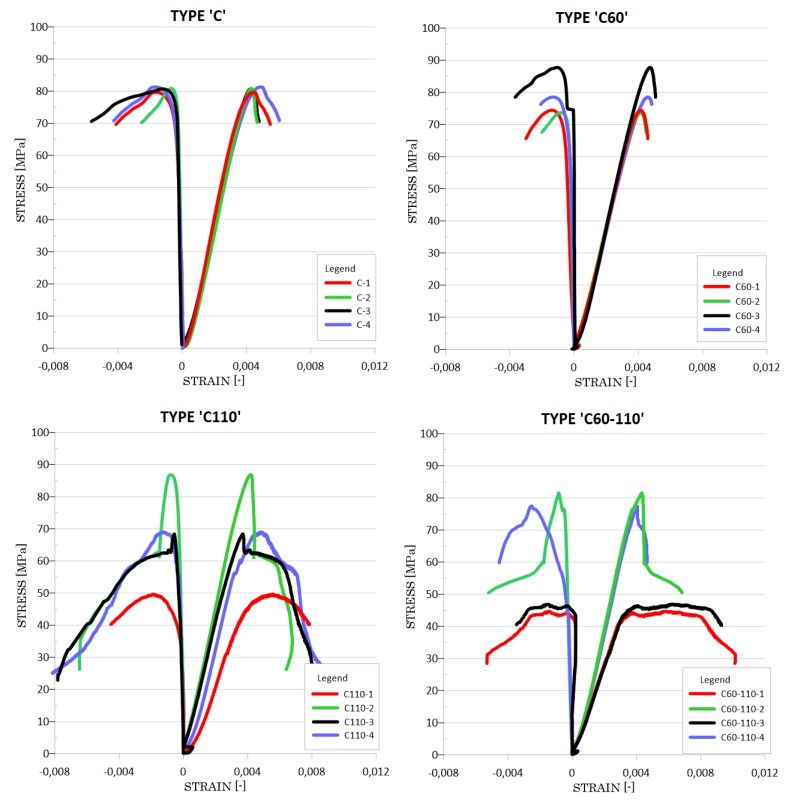

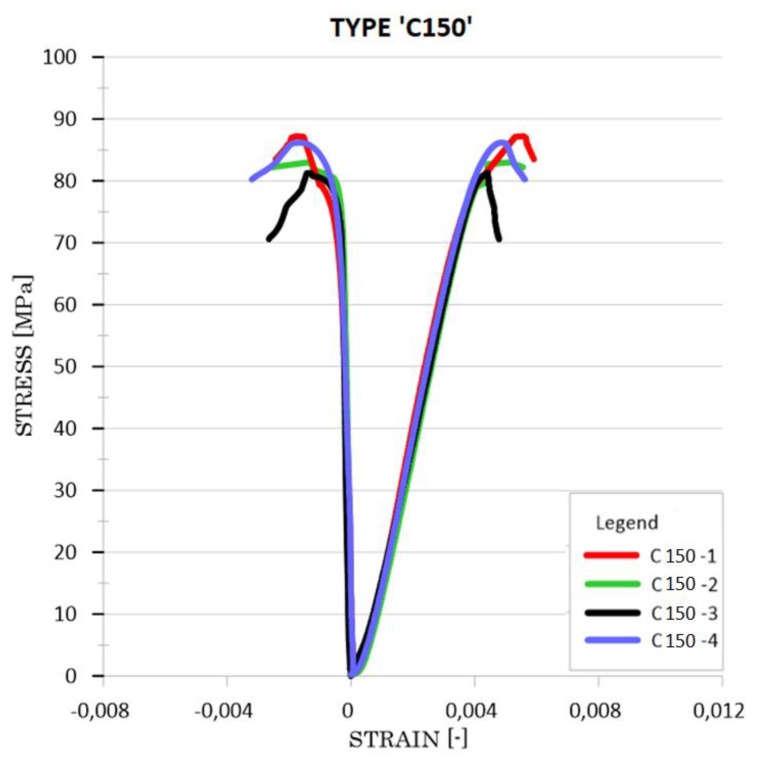
Stress-strain characteristics for the analyzed group of specimens.

The mechanical behavior of the HPSCFRC with the CFCR rings is presented using stress-strain curves. These relationships are shown in Figure 5. The vertical axis presents the axial stresses. The horizontal positive part of the graphs presents the axial strain, and in the negative part, the transverse strains are presented. In Table 4, in order to show the statistical parameters, selected properties of the tested specimens are shown. Based on the results, the stress-strain course for the HPSCFRC is linear elastic with a quasi-plastic range of work. This typical course is connected with the presence of steel fibres. A characteristic for this concrete is that after exceeding the maximum compressive stress, despite the violation of its structure, there is no rapid destruction of the concrete. A small but significant stretch of plastic flow is noticeable. The average compressive strength of the HPSCFRC is equal to 81.04 MPa, with a very small standard deviation equal to only 0.32 MPa. This indicates that both the production and laying of the concrete mix went flawlessly. The average axial and transverse strains of the HPSCFRC was 4.48 and 1.30, respectively. After exceeding the quasi-plastic range, propagation of the brittle-plastic destruction of the concrete can be observed. 

In the case of the concrete with one 60 mm CFCR ring inside, a similar stress-strain to the HPSCFRC was observed. The average maximum strength of the C60 was determined at a level of 78.62 MPa, which is nearly 3% lower than the HPSCFRC. This is also within the limits of 5% for statistical error. The main difference concerns the standard deviation, in the case of the C60 it is equal to 6.47 MPa. In reference to the average axial and transverse strains, the results are almost identical to the HPSCFRC. However, the only difference is the behavior of the C60 after exceeding the maximum compressive stress. The stress-strain characteristic is also brittle-plastic in this case, but more fragile than in the case of the HPSCFRC. The C110 group of specimens is characterized by a significant standard deviation of average compressive strength, which is equal to 15.31 MPa. In this case, a 15.66% lower compressive strength was observed. After reaching the maximum load capacity, a steeping loss of stiffness was noted. By using two CFCR rings with a 60 and 110 mm diameter, the average compressive strength has the lowest value. In the case of C60-110 specimens, the maximum standard deviation, average axial and transverse strains were noted. The stress-strain characteristics are different within the same type of samples. The behavior of the two samples is linear-elastic before maximum compressive stresses are obtained, plastic until the average axial strain is equal to 0.008, and fragile-plastic after exceeding this value. For these two samples, the maximum bearing capacity does not exceed the value of 50 MPa. These characteristics are different to the other two specimens, which are similar to the C60 group. During the forming of concrete structures, and especially in thin elements, the wall effect could be observed [38]. This phenomenon could lead to disorders such as a lower cement mortar content in the concrete mixture. A significant standard deviation of part of the specimens could be involved with this effect. The average Young’s modulus value is similar for all the analyzed groups. The highest average value was observed in the case of the C110 group and is equal to 37.65 GPa. When considering HPSCFRC reinforced with one external layer of CF sheets using cement matrix (C150 group), it could be seen that the compressive strength is similar to that of plain concrete. The growth of the average compressive strength by only 4.01% is within the range of statistical error. In this case, a lower average transverse strain during fracture was noted with regard to the reference samples. The average axial strain and stiffness are slightly higher when compared to group ‘C’. Depending on the stress-strain characteristic of each specimen, a variable value of stiffness was noted.

### 4.2. Course of Destruction

**Figure 6:**
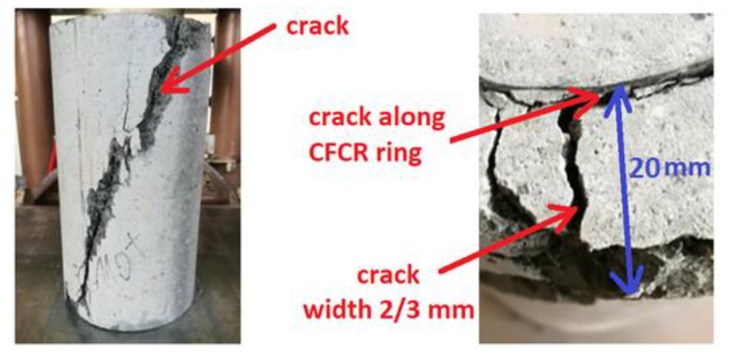
Typical failure mode of the HPSCFRC (high-performance self-compacting fibre-reinforced concrete) with a CFCR ring.

**Figure 7:**
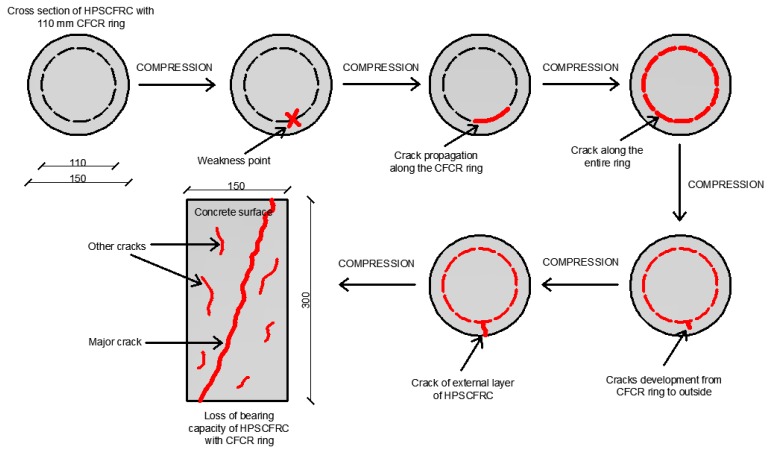
Observed stages of representative destruction progress on the example of the concrete with a CFCR ring in the uniaxial compression test.

The typical representative failure modes of the specimens with CFCR rings are presented in Figure 6. All the specimens failed without explosive rupture, which is in contrast to the classic behavior of concrete elements reinforced with a CFRP layer on their surface. As is shown in Figure 5, in the case of the specimens with CFCR rings, a big vertical crack from the top to the bottom of the specimen occurred. It was observed that the crack propagates from the outer contact surface between the concrete and the CFCR ring. As the load increases, a larger network of cracks forms within the concrete structure, which, in turn, leads to the destruction of the samples. Then, between the CFCR ring and the outer layer of the concrete, cracks are formed which propagate from the surface of the CFCR ring towards the outside. At the time of destruction, numerous cracks can be observed on the surface of the samples, of which one to two major cracks can be distinguished. This type of destruction is caused by a lack of appropriate adhesion between the CFCR rings and the HPSCFRC. Due to this fact, the shear forces on the outer surface of the CFCR rings lead to destruction of the outer part of the concrete, and consequently, to the loss of bearing capacity. It is worth emphasizing that due to the presence of dispersed steel reinforcement, all samples remained consistent. This is due to the good adhesion between the reinforcement and high-performance self-compacting concrete (HPSCC) and the small dimensions of the reinforcement, which improves the adhesion [39]. The stages of the destruction process in the uniaxial compression test on the example of the specimen ‘C110’ type are presented in Figure 7. A similar destructive process, as in the case of the other groups of concrete with CFCR rings, was observed.

**Figure 8:**
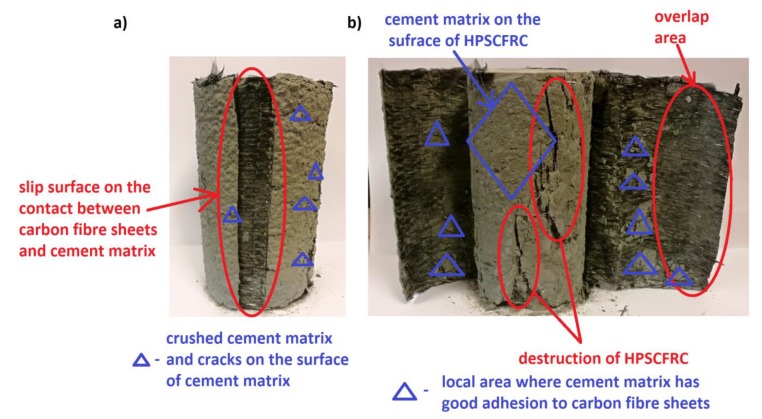
Typical failure mode of HPSCFRC reinforced with one layer of CF sheets using cement matrix: slip surface (**a**) and detached carbon fibres from the concrete surface (**b**).

In order to better explain the phenomenon of the impact of CFCR rings on the stress-strain capacity of HPSCFRC, determination of its behavior during the destruction of concrete reinforced with outer CF sheets using cement matrix could be useful. Figure 8 presents the typical failure mode of these types of specimens. As can be seen, the slip surface on the contact between the CF sheets and cement matrix occurred (Figure 8a). Due to the increase in stress in the sample, and in particular the increase in transverse deformations, the overlap was cut down in these areas. The shear stress was higher than the adhesion between the CF sheets and cement matrix. On the surface of the specimens, a crushed cement matrix and cracks on the surface can be observed. Figure 8b presents more details regarding the efficiency of the contact. The carbon fibre sheets were not broken, and the structure of the HPSCFRC was destroyed. On part of the concrete surface, the cement matrix could be observed, which shows that the adhesion between the concrete and cement matrix was generally good. Good adhesive properties between the CF sheets and cement matrix occurred locally, in this case, the adhesion was higher than between the HPSCFRC and cement matrix (the cement matrix was broken away from the concrete surface). The cement matrix does not have the same properties and binding as epoxy resin, which leads to the lack of full filtration into the structure of carbon fibers with the analyzed binder. 

## 5. Conclusions

In the article, the influence of carbon fibre coated reinforcement rings on the behavior of high-performance self-compacting fibre-reinforced concrete was analyzed. Based on the experimental results, the following conclusions could be drawn:

- When using CFCR rings, the loss of the bearing capacity of concrete elements can be observed. With an increase in both the number of CFCR layers and the distance from the center of gravity of the specimens, the compressive strength decreases. In the case of using two rings inside the structure of the concrete, a 23% loss of compressive strength was observed.
- No cooperation between the CFCR rings and concrete was observed. The outer surface of the CFCR ring could be treated as the shear surface, which does not allow cooperation between the HPSCFRC and the CFRC rings. Cracks appear on the surface of the CFCR rings and then propagate to the external edges of the specimen, which in turn leads to their destruction.
- Usage of CFCR rings inside the structure of HPSCFRC is not justified due to the executive difficulties and stress-strain behavior for each analyzed configuration.
- The efficiency of reinforced HPSCFRC using outer CF sheets with cement matrix is not as sufficient as could be expected. Due to the observed low adhesion between the composite reinforcement and concrete in the ‘C150’ type specimens, it is not recommended to reinforce this type of concrete with CF sheets using infusible cement matrix.

For future research, particular microanalysis concerning the contact between CFRP and HPSCFRC, as well as numerical simulations, will be performed. This knowledge will allow for crucial aspects occurring in the internal structure of these types of elements to be determined. Moreover, the influence of the type of concrete surface (unprepared, grinded and sanded) on the properties of HPSCFRC reinforced with CF sheets using epoxy resin and cement matrix were performed, and will be published soon.

## Figures and Tables

**Table 1:** Properties of the carbon fibres.

Type	Young Modulus [GPa]	Tension Strength [MPa]	Ultimate Elongation at the Break [%]	Effective Thickness [mm]	Density [g/m^2^]
Sikawrap 301c	230	4900	1.7	0.167	304

**Table 2:** Proportions of the concrete mixture.

Cement [kg/m^3^]	Sika Fume [kg/m^3^]	Coarse Aggregate [kg/m^3^]	Fine Aggregate [kg/m^3^]	Super-Plasti-Cizer [kg/m^3^]	Steel Fibres [kg/m^3^]	Water [kg/m^3^]	W/C [-]
500	60	1000	650	17.5	78	160	0.32

**Table 3:** Analyzed types of specimens.

Type	Diameter of CFCR Ring [mm]
C	-
C60	60
C110	110
C60-110	60 and 110
C150	150 *

* CF (carbon fibre) was stuck to the concrete surface.

**Table 4:** Selected properties of the tested specimens.

Specimen	Compressive Strength [MPa]	Average Compressive Strength [MPa]	Standard Deviation [MPa]	Average Strength in Relation to reference [%]	Axial Strain during Fracture [-]	Average Axial Strain during Fracture [-]	Transverse Strain during Fracture [-]	Average Transverse Strain during Fracture [-]	Young Modulus [GPa]	Average Young Modulus [GPa]
C-1	81.31				4.51		1.64		34.67	
C-2	80.81	81.04	0.32	0	4.29	4.48	0.67	1.30	31.78	34.89
C-3	80.71				4.27		1.23		36.78	
C-4	81.31				4.86		1.64		36.31	
C60-1	74.44				4.16		1.39		34.77	
C60-2	73.70	78.62	6.47	−2.99	4.15	4.41	0.81	1.34	34.93	34.42
C60-3	87.78				4.72		1.06		35.65	
C60-4	78.54				4.61		1.33		34.34	
C110-1	49.32				5.83		2.17		30.36	
C110-2	86.82	68.35	15.31	−15.66	4.29	4.71	0.78	1.18	38.29	37.65
C110-3	68.39				3.80		0.57		43.20	
C110-4	68.86				4.90		1.18		38.75	
C60-110-1	44.40				6.49		1.65		28.05	
C60-110-2	81.53	62.49	19.73	−22.89	4.34	5.51	0.85	37.65	35.49	
C60-110-3	46.56				7.20		1.79		27.08	33.51
C60-110-4	77.45				4.02		2.55		43.40	
C150-1	87.11				4.63		1.56		36.45	
C150-2	82.76				4.42		1.05		32.48	
C150-3	81.35	84.29	2.69	4.01	4.56	4.58	1.12	1.23	35.15	35.01
C150-4	85.94				4.71		1.18		35.97	
